# LOVD–DASH: A comprehensive LOVD database coupled with diagnosis and an at‐risk assessment system for hemoglobinopathies

**DOI:** 10.1002/humu.23863

**Published:** 2019-09-11

**Authors:** Li Zhang, Qianqian Zhang, Yaohua Tang, Peikuan Cong, Yuhua Ye, Shiping Chen, Xinhua Zhang, Yan Chen, Baosheng Zhu, Wangwei Cai, Shaoke Chen, Ren Cai, Xiaoling Guo, Chonglin Zhang, Yuqiu Zhou, Jie Zou, Yanhui Liu, Biyan Chen, Shanhuo Yan, Yajun Chen, Yuehong Zhou, Hongmei Ding, Xiarong Li, Dianyu Chen, Jianmei Zhong, Xuan Shang, Xuanzhu Liu, Ming Qi, Xiangmin Xu

**Affiliations:** ^1^ Department of Medical Genetics Southern Medical University Guangzhou Guangdong China; ^2^ Guangdong Technology and Engineering Research Center for Molecular Diagnostics of Human Genetic Diseases Guangzhou Guangdong China; ^3^ Guangdong Key Laboratory of Biological Chip Guangzhou Guangdong China; ^4^ DIAN Diagnostics Hangzhou China; ^5^ Institute for Basic Medical Sciences, Westlake Institute for Advanced Study Hangzhou Zhejiang China; ^6^ BGI Clinical Laboratories‐Shenzhen Shenzhen Guangdong China; ^7^ Department of Hematopathology 303rd Hospital of the People's Liberation Army Nanning Guangxi China; ^8^ Department of Pediatrics Affiliated Hospital of Zunyi Medical University Zunyi China; ^9^ Genetic Diagnosis Center First People's Hospital of Yunnan Province Kunming Yunnan China; ^10^ School of Basic Medicine Hainan Medical University Haikou Hainan China; ^11^ Department of Pediatrics Guangxi Zhuang Autonomous Region Women and Children Health Care Hospital Nanning Guangxi China; ^12^ Department of Medical Genetics Liuzhou Municipal Maternity and Child Healthcare Hospital Liuzhou Guangxi China; ^13^ Department of Pediatrics Maternity and Child Health Care Hospital of Foshan City Foshan Guangdong China; ^14^ Department of Clinical Laboratory Guilin Women and Children health care hospital Guilin Guangxi China; ^15^ Department of Clinical Laboratory Zhuhai Municipal Maternity and Child Healthcare Hospital Zhuhai Guangdong China; ^16^ Department of Clinical Laboratory Maternal and Child Health Hospital in Meizhou Meizhou Guangdong China; ^17^ Department of Prenatal Diagnosis Center Maternal and Child Health Hospital Dongguan Guangdong China; ^18^ Department of Clinical Laboratory Baise Women and Children Care Hospital Baise Guangxi China; ^19^ Department of Clinical Laboratory Qinzhou Maternal and Child Health Hospital Qinzhou Guangxi China; ^20^ Center For Prenatal Diagnosis Shaoguan Shaoguan Municipal Maternity and Child Healthcare Hospital Guangdong China; ^21^ Department of Clinical Laboratory The People's Hospital of Yunfu City Yunfu Guangdong China; ^22^ Department of Clinical Laboratory Pingguo Women and Children Care Hospital Baise Guangxi China; ^23^ Beijing GeneDock Technology Company Beijing China; ^24^ School of Basic Medical Sciences Zhejiang University Hangzhou Zhejiang China; ^25^ Center for Genetic & Genomic Medicine, JamesWatson Institute of Genome Sciences Zhejiang University Medical School 1st Affiliated Hospital Hangzhou Zhejiang China

**Keywords:** clinical genotyping, DASH, database, hemoglobinopathy, LOVD, molecular screening

## Abstract

Hemoglobinopathies are the most common monogenic disorders worldwide. Substantial effort has been made to establish databases to record complete mutation spectra causing or modifying this group of diseases. We present a variant database which couples an online auxiliary diagnosis and at‐risk assessment system for hemoglobinopathies (DASH). The database was integrated into the Leiden Open Variation Database (LOVD), in which we included all reported variants focusing on a Chinese population by literature peer review‐curation and existing databases, such as HbVar and IthaGenes. In addition, comprehensive mutation data generated by high‐throughput sequencing of 2,087 hemoglobinopathy patients and 20,222 general individuals from southern China were also incorporated into the database. These sequencing data enabled us to observe disease‐causing and modifier variants responsible for hemoglobinopathies in bulk. Currently, 371 unique variants have been recorded; 265 of 371 were described as disease‐causing variants, whereas 106 were defined as modifier variants, including 34 functional variants identified by a quantitative trait association study of this high‐throughput sequencing data. Due to the availability of a comprehensive phenotype‐genotype data set, DASH has been established to automatically provide accurate suggestions on diagnosis and genetic counseling of hemoglobinopathies. LOVD‐DASH will inspire us to deal with clinical genotyping and molecular screening for other Mendelian disorders.

## 1 INTRODUCTION

Hemoglobinopathies are the most common monogenic disorders worldwide. The major β‐hemoglobinopathies, especially sickle cell disease and β‐thalassemia are lethal hemoglobinopathies that have caused global health burdens due to their serious pathogenicity and high prevalence (Taher, Weatherall, & Cappellini, 2018; Weatherall., 2010). Previous efforts and programs for preventing hemoglobinopathies have proved to be effective in the Mediterranean populations, especially for β‐thalassemia, showing a reduction from 1:250 live births to 1:1660 in 2009 (Cao & Kan, 2013). The birth rates of hemoglobinopathies, however, remain high. In China, approximately 12,900 newborns are estimated with hemoglobin disorders of various types each year, which in turn may cause a serious social burden (Shang et al., 2017; Xiong et al., 2010). The number of fetuses with hemoglobinopathies can be largely reduced if a robust system for clinicians is developed to master standard guidelines and to rapidly make correct clinical management choices for hemoglobinopathy patients or at‐risk couples.

Previous observations involving hemoglobin switching have shown that elevated fetal hemoglobin expression ameliorates the severity of β‐hemoglobinopathies (Vinjamur, Bauer, & Orkin, 2017). Thus, an accurate diagnosis of hemoglobinopathies calls for not only the proper genotyping of the disease‐causing mutations in globin gene clusters, but also newly identified variants in modifier genes, such as *KLF1*, *BCL11A*, and *GATA1*, which are responsible for altered expression of γ‐globin and also influence β‐thalassemia severity (Bauer & Orkin, 2015; D. Liu et al., 2014; Thein et al., 2007). Substantial effort has been made to establish the databases to record global mutation spectra causing and modifying hemoglobinopathies (Giardine et al., 2011; Kountouris et al., 2014). HbVar, built by Giardine et al. (Hardison et al., 2002) has thus far been an authoritative hemoglobinopathy database for both researchers and clinicians. We present herein a comprehensive variant database of hemoglobinopathies focusing on a Chinese population, recording the details of all reported variants through literature peer review‐curation and existing databases. Moreover, unpublished data from our laboratory, including all the phenotype–genotype datasets derived from high‐throughput sequencing data of 2,087 hemoglobinopathy patients and 20,222 general southern Chinese individuals, were also merged into the database (Shang et al., 2017). The addition of 34 novel functional variants from these genes has been detected using this high‐throughput approach. All the variants are classified according to American College of Medical Genetics and Genomics (ACMG) recommendations with the use of specific standard terminology: “pathogenic”, “likely pathogenic”, “uncertain significance”, “likely benign”, and “benign”. (Richards et al., 2015; Table S1). Details of all the variants are integrated into the Leiden Open Variation Database, which is available at http://www.genomed.zju.edu.cn/LOVD3/genes.

An online auxiliary diagnosis and at‐risk assessment system for inherited hemoglobinopathy (DASH) has also been established based on the following: (a) the integrity of the hemoglobinopathy mutation spectrum of a Chinese population; (b) the availability of a comprehensive phenotype‐genotype data set corresponding to the 22,309 samples; and (c) the detailed information of variants according to the latest version of HbVar (Giardine et al., 2014). Aiming to accomplish the molecular screening and clinical genotyping of hemoglobinopathies in a Chinese population, DASH consists of three main workflows. DASH not only infers the thalassemia trait based on the input of the hematologic phenotype but also recognizes the uploaded copy number variants (CNVs) and single nucleotide variants (SNVs) data then interprets the data with a specific hemoglobinopathy annotation library. Both disease‐causing and modifier variants will be evaluated for a combined analysis, which will ultimately lead to an overall hemoglobinopathy diagnosis. Furthermore, the system will conduct an at‐risk assessment of known disease‐causing mutations and reveal critical clinical information for potential offspring. A diagnostic and assessment report will be automatically presented which could provide accurate suggestions on diagnosis and genetic counseling of hemoglobinopathies. DASH is available at http://www.smuhemoglobinopathy.com.

In this study, we portrayed the most comprehensive mutation spectrum of hemoglobinopathies in the Chinese population. In addition, LOVD‐DASH will make a contribution in research and clinical application and provide a new method for treatment and precaution of hemoglobinopathies in Chinese patients. With the LOVD database and DASH system, we are one step closer to complete molecular screening and accurate clinical genotyping of hemoglobinopathies.

## 2 METHODS AND RESULTS

### 2.1 LOVD database of hemoglobinopathy variants in a Chinese population

#### 2.1.1 Data collection and database content

The bulk of data on gene variants was derived from published data, including databases (HbVar: http://globin.cse.psu.edu/; IthaGenes: http://www.ithanet.eu/db/ithagenes), literature mining from PubMed (https://www.ncbi.nlm.nih.gov/pubmed), and Chinese core journals (http://xueshu.baidu.com/). The other source is unpublished data from our laboratory, including phenotype‐genotype datasets derived from high‐throughput sequencing data of 2,087 patients with hemoglobinopathy and 20,222 general previously mentioned southern Chinese individuals. The study was approved by the Medical Ethics Committee in accordance with the Declaration of Helsinki. All the clinical data of participants have been obtained and curated under agency ethical guidelines. Variations in globin genes (*HBA1,* MIM# 141800; *HBA2,* MIM# 141850; *HBB,* MIM# 141900; *HBG1,* MIM# 142200; *HBG2,* MIM# 142250; *HBD,* MIM# 142000) and nonglobin genes (*BCL11A,* MIM# 606557; *KLF1,* MIM# 600599; *GATA1,* MIM# 305371), as well as the intergenic region of *HMIP* (MIM# 142470), were categorized into disease‐causing and modifiers, and would be accepted as keywords to perform literature mining on hemoglobinopathies. Currently, 371 unique variants have been recorded; 265 of the 371 unique variants were described as disease‐causing variants, while 106 were defined as modifier variants (Table 1), including 34 functional variants identified by a quantitative trait association study of the high‐throughput sequencing data in Plink (Figure S1; Table 2; Table S2). The inevitable bias caused by haplotype that the causative variant(s) among them cannot be judged simply by statistical approaches. For further research, the corresponding phenotypic information and genotypic data, including disease‐causing and identified modifier variants in detail of 2,087 hemoglobinopathy cohort were displayed in http://www.genomed.zju.edu.cn/LOVD3/individuals.

**Table 1:** Summary of disease‐causing and modifier variants of hemoglobinopathies in LOVD‐China

Disease‐causing variants						
	*HBB*	*HBA1*	*HBA2*	*HBD*	*HBG1* ^a^	*HBG2* ^a^
OMIM	141900	141800	141850	142000	142200	142250
Location	11p15.4	16p13.3	16p13.3	11p15.4	11p15.4	11p15.4
Pathogenicity						
Hb variant	28	6	13	4	2	2
Thalassemia	92	27	33	17	0	0
HPFH^b^	2	0	0	2	1	0
Uncertain significance	2	11	8	2	8	5
Total	124	44	54	25	11	7

Modifier variants						
	*KLF1* ^c^	*BCL11A*	*HMIP*	*GATA1* ^d^	*HBG1*	*HBG2*
OMIM	600599	606557	142470	305371	142200	142250
Location	19p13.13	2p16.1	6q22.3–23.1	Xp11.23	11p15.4	11p15.4
Modification						
Elevated Hb F Level	18	3	17	0	5	4
Decreased Hb F Level	0	9	3	0	4	7
Uncertain significance	1	1	24	7	0	3
Total	19	13	44	7	9	14

*Note*: All the variants are classified according to ACMG recommendations. The pathogenicity was described as Hb variant, thalassemia, and hereditary persistence fetal hemoglobin (HPFH). “Hb variant” includes not only variants result in clinical significance, but all the reported abnormal hemoglobin variants. Modifier variants refer to all the collectible variants reported to be HbF‐related or be significant in influencing the severity of hemoglobinopathies in the Chinese population. Variants in disease‐causing genes without known pathogenicity were defined as the variants with “uncertain significance” (VUS). “Uncertain significance” in modifier variants, especially in *HMIP* region, refers to the variants reported to be HbF‐related but with unclear modification.

Abbreviations: ACMG, American College of Medical Genetics and Genomics; LOVD, LOVD, Leiden Open Variation Database; OMIM, Online Mendelian Inheritance in Man

^a^ Appropriately, *HBG1* and *HBG2* genes can be classed as modifier genes. “Hb variant” in these genes referred to the variants which lead to abnormal fetal hemoglobin such as Hb F‐Jiangsu (*HBG1*:c.403G>A).

^b^ HPFH involves only the deletion forms.

^c^ Although bulks of modifier variants have been detected in non‐globin genes like *BCL11A* or *HMIP* region etc., functional variants within *KLF1* have the most clinical significant influence on the severity of β‐hemoglobinopathies in Chinese population.

^d^ Variants in erythroid transcription factors *GATA1* are reported to be related with HbF, HbA2, and severity of hemoglobinopathies.

**Table 2:** Functional variants from 22,309 high‐throughput sequencing data

SNPs	Locus	Location^a^	Nucleotide change	Frequency	Modification	*P*‐value	HbF level of carriers(g/L)	HbF level of non‐carriers(g/L)
rs61749494	*BCL11A*	2:60689441	T>C	0.251	Elevated HbF	4.24 × 10^−6^	18.6820	11.6439
rs10189857	*BCL11A*	2:60713235	A>G	0.9235	Decreased HbF	1.64 × 10^−6^	12.8591	20.0668
rs6545816	*BCL11A*	2:60714861	A>C	0.9216	Decreased HbF	3.24 × 10^−6^	12.8711	19.7456
rs1427407	*BCL11A*	2:60718043	T>G	0.9275	Decreased HbF	1.95 × 10^−6^	12.9450	19.3588
rs7599488	*BCL11A*	2:60718347	C>T	0.9235	Decreased HbF	2.77 × 10^−6^	12.8542	20.1268
rs766432	*BCL11A*	2:60719970	C>A	0.9255	Decreased HbF	1.65 × 10^−5^	13.0060	18.4325
rs4671393	*BCL11A*	2:60720951	A>G	0.9275	Decreased HbF	3.43 × 10^−6^	12.9880	18.8090
rs375867652	*HMIP*	6:135419038	delC	0.3275	Elevated HbF	1.59 × 10^−4^	17.3037	11.5147
rs11759553	*HMIP*	6:135422296	A>T	0.3451	Elevated HbF	5.61 × 10^−5^	17.2904	11.3657
rs35959442	*HMIP*	6:135424179	C>G	0.349	Elevated HbF	6.06 × 10^−5^	17.2321	11.3613
rs4895440	*HMIP*	6:135426558	A>T	0.349	Elevated HbF	6.06 × 10^−5^	17.2321	11.3613
rs4895441	*HMIP*	6:135426573	A>G	0.349	Elevated HbF	6.06 × 10^−5^	17.2321	11.3613
rs9402686	*HMIP*	6:135427817	G>A	0.351	Elevated HbF	6.06 × 10^−5^	17.1452	11.3906
rs9494142	*HMIP*	6:135431640	T>C	0.3627	Elevated HbF	5.17 × 10^−5^	16.9296	11.4070
rs6934903	*HMIP*	6:135451564	T>A	0.3373	Elevated HbF	9.34 × 10^−5^	16.6348	11.7694
rs78981054	*HBG1*	11:5270347	delAAAG	0.9863	Decreased HbF	4.66 × 10^−8^	13.1293	33.6006
rs34879481	*HBG2*	11:5274452	insT	0.1392	Elevated HbF	4.19 × 10^−12^	23.3682	11.7998
rs28379094	*HBG1*	11:5269806	C>T	0.9843	Decreased HbF	1.65 × 10^–12^	13.0232	37.7005
rs2187608	*HBG1*	11:5269931	G>C	0.1373	Elevated HbF	1.41 × 10^–12^	23.3314	11.832
rs7482933	*HBG1*	11:5270002	G>A	0.8588	Decreased HbF	1.15 × 10^–9^	11.8743	22.7546
rs2855039	*HBG1*	11:5271671	C>T	0.1373	Elevated HbF	6.93 × 10^–12^	23.3314	11.832
rs2855038	*HBG1*	11:5272154	T>C	0.9863	Decreased HbF	4.46 × 10^–11^	13.1293	33.6006
rs2855036	*HBG1*	11:5272682	C>T	0.1373	Elevated HbF	1.30 × 10^–11^	23.3314	11.832
rs2070972	*HBG2*	11:5274717	A>C	0.9843	Decreased HbF	5.86 × 10^–11^	13.1411	30.303
rs11036474	*HBG2*	11:5275178	T>C	0.1412	Elevated HbF	7.90 × 10^–12^	23.3499	11.7764
rs11036475	*HBG2*	11:5275240	G>A	0.9863	Decreased HbF	2.06 × 10^–11^	13.1293	33.6006
rs11036476	*HBG2*	11:5275343	C>T	0.9863	Decreased HbF	9.69 × 10^–11^	13.1293	33.6006
rs2070973	*HBG2*	11:5275407	T>C	0.9863	Decreased HbF	9.69 × 10^–11^	13.1293	33.6006
rs7482144	*HBG2*	11:5276169	G>A	0.1412	Elevated HbF	1.81 × 10^–11^	23.3499	11.7764
rs2855123	*HBG2*	11:5277078	A>T	0.9863	Decreased HbF	3.92 × 10^‐10^	13.1293	33.6006
rs2855122	*HBG2*	11:5277236	C>T	0.9863	Decreased HbF	1.20 × 10^–11^	13.1293	33.6006
rs2855121	*HBG2*	11:5277291	C>T	0.1392	Elevated HbF	7.90 × 10^–12^	23.3682	11.7998
rs34306743	*HBG1*	11:5272553	insA	0.1373	Elevated HbF	1.30 × 10^–11^	23.3314	11.832
rs483352838	*KLF1*	19:12996518	insGGCGCCG	0.0137	Elevated HbF	1.93 × 10^−6^	39.2343	13.0509

*Note*: Among the 510 β^0^/β^0^ samples from 22,309 sequencing data, 74 variants were shown to be significant after association analysis in Plink judged by the *P*‐values after a Bonferroni correction. 34 of the variants were located in our candidate genes or the *HMIP* region. The 74 variants are available in the supplementary document.

Abbreviation: SNP, single nucleotide polymorphism.

^a^ The chromosomal locations are given in GRCh37/hg19 coordinates.

#### 2.1.2 Database structure

**Figure 1:**
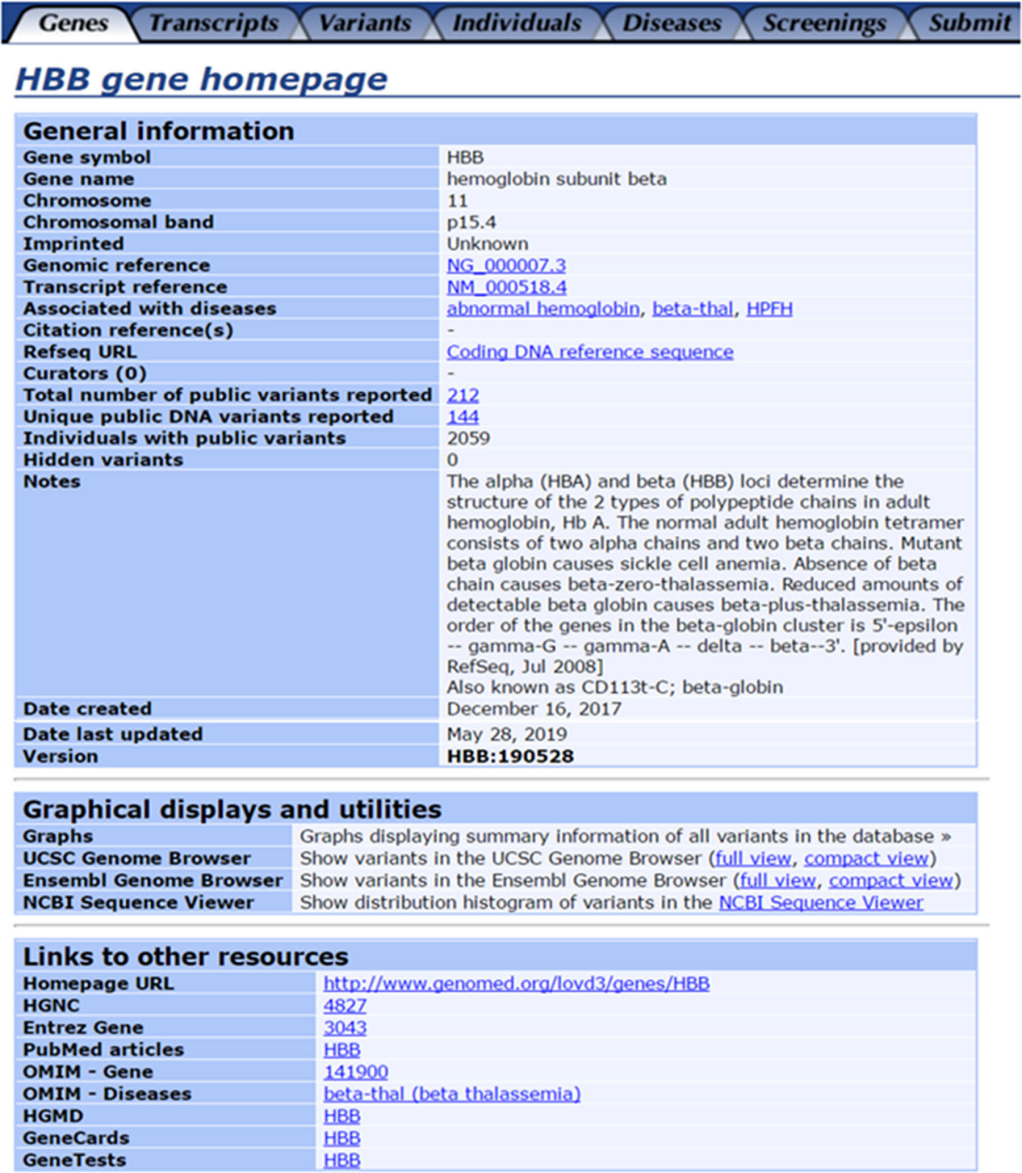
Homepage of the *HBB* gene from our LOVD‐China database. LOVD, Leiden Open Variation Database

Taking *HBB* as an example (Figure 1), all the datasets are classified and presented in different columns including genes, transcripts, variants, individuals, diseases, and so forth. For instance, “individuals” column contains the basic information, genotypes, and phenotypes of all individuals with variants in the *HBB* gene. Besides, the homepage of the variant database consists of the following three sections: (a) The general information section contains basic information about the *HBB* gene. The reference sequences of *HBB* can be obtained in this section. The entries of public variants can be found in the “total number of public variants reported” listing. Detailed information of variants, such as DNA change, protein change, variant type, location, and the information of patients are available for download; (b) The graphical display section offers diagrams to show summary information of all variants in the database; (c) The linkage section shows other authoritative resources, the including HUGO Gene Nomenclature Committee (https://www.genenames.org/), Entrez gene (http://www.ncbi.nlm.nih.gov/gene/), Online Mendelian Inheritance in Man (OMIM; http://www.omim.org), the Human Gene Mutation Database (HGMD; http://www.hgmd.cf.ac.uk/), Genecards (http://www.genecards.org/), and the Genetic Testing Registry (GTR; https://www.ncbi.nlm.nih.gov/gtr/).

#### 2.1.3 Data submission

The LOVD‐China database is available for public submission. The submitter should register and sign in before submission. The literature or evidence which can support the clinical significance of the variants is required. Submitters should complete the variant data and corresponding phenotypic information in detail as much as possible. It is necessary that all variant data submitted should be named according to the nomenclature of the Human Genome Variation Society (HGVS; den Dunnen et al., 2016). The authors of this study are responsible for the manual curation of the database by checking each entry, adding new entries, and updating both existing variations and epidemiologic data. A more detailed introduction can be found in the documentation at http://www.genomed.zju.edu.cn/LOVD3/docs/.

### 2.2 Diagnosis and at‐risk assessment system of hemoglobinopathies

#### 2.2.1 DASH structure

**Figure 2:**
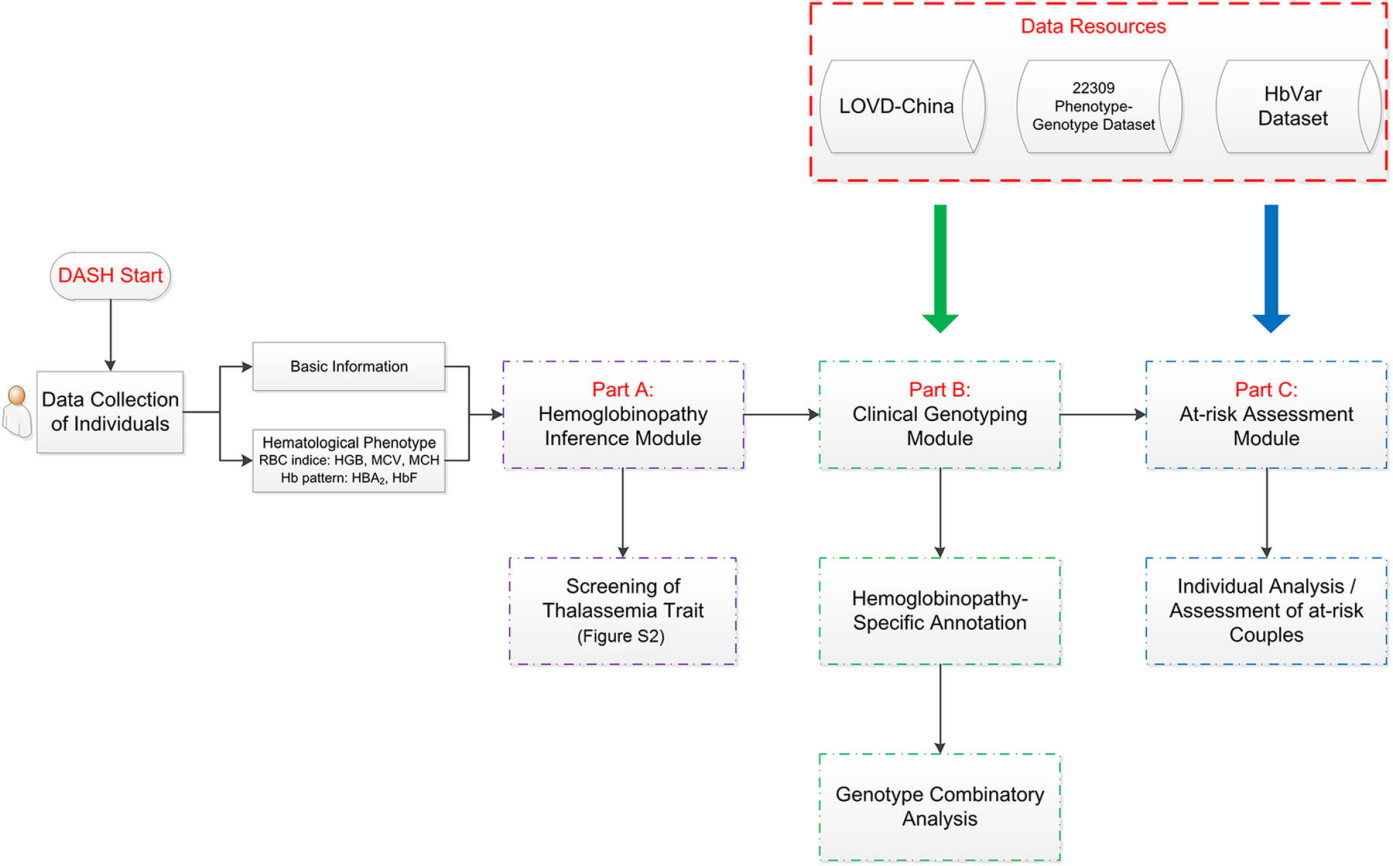
Workflow of DASH. Data resources including LOVD‐China, 22,309 phenotype‐genotype data set, and HbVar data set were mainly used for the clinical genotyping module and at‐risk assessment module. DASH, diagnosis and at‐risk assessment system for hemoglobinopathies; LOVD, Leiden Open Variation Database

We integrated LOVD‐China, the 22,309 phenotype‐genotype individual data set, and HbVar data as a comprehensive hemoglobinopathy‐specific annotation data set for DASH (Figure 2). DASH is freely accessible online where three main workflows can be chosen from the homepage, including the hemoglobinopathy inference module, the clinical genotyping module, and the at‐risk assessment module.

#### 2.2.2 DASH for hemoglobinopathy inference

The hemoglobinopathy inference module provides a judgment algorithm according to the traditional routine strategy for thalassemia carrier screening (Traeger‐Synodinos et al., 2015). Hematologic and biochemical tests and subsequent molecular genetic testing are required for identification (Danjou et al., 2015). Thus, both basic information and hematologic phenotype are required as the input. Basic information includes age, gender, and native places, while hematologic phenotype includes red blood cell indices (HGB, MCH, MCV, and Hb pattern [Hb A_2_ and Hb F]). A standard criterion was used for the judgment of the thalassemia trait (Figure S2). For example, a 5‐year‐old girl from Guangdong province of China had the following hematologic phenotype: MCH, 24 pg; MCV, 73 fL; Hb F, 3%; and Hb A_2_, 6% (http://www.genomed.zju.edu.cn/LOVD3/individuals/00001639) will be inferred as the β‐thalassemia trait in the output report. In addition, clinical genotyping is highly recommended for this individual; however, individuals with “silent” forms of thalassemia are undetectable because such individuals have normal or borderline red cell indices and/or Hb A_2_ levels (Hallam et al., 2014). Moreover, it is important to note that iron deficiency alone or co‐exist with the thalassemias can also cause microcytic hypochromic anemia, which could lead to misinterpretation. If an individual is found to be iron‐deficient, it is recommended to repeat the hematologic screen once the individual is iron‐replete (Traeger‐Synodinos et al., 2015).

#### 2.2.3 DASH for clinical genotyping

The clinical genotyping module consists of two sub‐modules (hemoglobinopathy‐specific annotation and genotype combinatory analysis), which will be executed sequentially. Different format of hemoglobinopathy‐related SNVs and CNVs list can be recognized as inputs, then annotated by the integrated comprehensive hemoglobinopathy‐specific annotation data set. After annotation, disease‐causing and modifier variants will be evaluated for a combined analysis, which ultimately leads to an overall hemoglobinopathy diagnosis, especially for β‐thalassemia. For example, heterozygotes of the β‐variant combined with α‐globin gene triplicates or quadruplicates will be reported as β‐thalassemia intermedia and compound heterozygotes or homozygotes of variants located in or destroying the zinc finger domain of the *KLF1* gene will be reported as atypical thalassemia. For perfect use of this module, the details of the input requirement are on the right side and output interpretation can be obtained in the Q&A from the homepage (http://www.smuhemoglobinopathy.com/question/#tab=1).

#### 2.2.4 DASH for at‐risk assessment

At‐risk assessment module has been established for couples, the variant list from individual and spouse are required. Format and examples of variants are provided in the right side of the module. For each individual, clinical genotyping will be performed first to get an overall hemoglobinopathy diagnosis, then combinatory analysis of variants of individual and spouse will report whether or not the offspring of this couple will be at‐risk for hemoglobin disorders. The possible at‐risk genotype and modifier variants of offspring will be reminded to assist the clinicians for comprehensive genetic counseling.

## 3 DISCUSSION

The LOVD‐China database, which was first built by Zhejiang University as part of the International Human Variome Project, has properly managed and stored thousands of phenotype‐genotype datasets from China in strict accordance with the regulation of Ministry of Science and Technology of PR China, since 2008 (Burn & Watson, 2016). The LOVD‐China database has already embodied comprehensive mutation spectra and the phenotypic impact of several diseases, including breast cancer, colorectal cancer, and LQTS (Pan et al., 2011; Zhang et al., 2010). In this study, we established the LOVD‐China database for hemoglobinopathies by integrating all the collectible variants corresponding to the candidate genes focusing on a Chinese population, especially 74 functional modifier variants identified from our local 2,087 hemoglobinopathies samples which will make efforts to provide a new target for precise diagnosis of the clinical severity β‐thalassemia. In addition, we developed and validated a DASH aimed to help facilitate the accurate diagnosis and counseling from the results of both traditional approaches and molecular screening of hemoglobinopathies in Chinese. Six globin genes (*HBB*, *HBA1*, *HBA2*, *HBD*, *HBG1*, and *HBG2*) and three nonglobin modifiers (*KLF1*, *BCL11A,* and *HMIP*), which have been shown to possess the greatest efficacy to statistically explain the modest clinical symptoms of β‐thalassemia, were considered as our candidate genes. Notably, other modifier genes of hemoglobinopathies exist which may be relevant to the phenotype of patients. The aim of this study, however, was to portray the most comprehensive mutation spectrum of hemoglobinopathies in a Chinese population and provide a new method for automatic auxiliary diagnosis of hemoglobinopathies.

There is an all‐time difficulty to make an accurate determination of the morbidity for hemoglobinopathies mainly due to complicated environmental factors, medical conditions, and individual difference. Besides, as mentioned before, modifier genes have a significant impact on the severity of hemoglobinopathies. Combination analysis of disease‐causing and modifier variants is considered to be the key factor underlying accurate clinical genotyping (Danjou et al., 2015). During the era of next‐generation sequencing, the bulk of variants and polymorphisms have been identified in the hemoglobinopathy‐relevant genes, especially in modifier genes, such as the *KLF1*, *BCL11A*, and *HMIP* regions (Basak & Sankaran, 2016; Orkin, 2016). More and more variants in these genes were shown to be clinically effective, while the contributions towards clinical severity have shown great ethnic‐specificity. The reawakening of fetal hemoglobin based on these variants holds promise for new therapies for β‐hemoglobinopathies (Bauer, Kamran, & Orkin, 2012). Our group has accumulated various kinds of samples representing different combinations of disease‐causing variants and modifiers. For example, the *KLF1* gene plays an important role in alleviating the clinical severity of β‐thalassemia (D. Liu et al., 2014; Tepakhan et al., 2016). As the cases in Table 3 show, patients with the β^0^/β^0^ genotype, which may result in thalassemia major, turns out to be thalassemia intermediate when they carry functional variants in *KLF1*. Also, alpha multi‐copies are considered to be modifier variants. The heterozygote of β‐thalassemia is asymptomatic, whereas the heterozygote of β‐thalassemia, combined with alpha multi‐copies, results in a thalassemia intermediate phenotype (Mettananda, Gibbons, & Higgs, 2015; Table 3 and Table S3). Thus, the combined analysis module of DASH is necessary, especially for the accurate clinical genotyping of these samples.

**Table 3:** 26 complicated cases of compound heterozygotes in combination with modifier variants

Major classes	Genotypes	Number of patients	
		TM	TI
β‐thal modification samples		4	19
α‐Globin gene triplication	(β^0^/β^0^, ααα/αα)	3	0
	(β^0^/β^+^, ααα/αα)	1	0
	(β^0^/β^N^ or β^+^/β^N^, ααα/αα or ααα/ααα)	0	9
Other modifier variants	(β^0^ / β^0^) + (*KLF1* ^M^/*KLF1* ^N^)	0	5
	(β^0^ / β^0^) + 4 significant variants*	0	5
Atypical thalassemia samples		**0**	**3**
Microcytic hypochromic anemia	(*KLF1* ^M^/*KLF1* ^M^)	0	3

*Note*: Four significant variants*: 4 functional variants exerting a significant impact on the clinical severity of β‐thalassemia patients: *HBA1* and *HBA2* disease‐causing variants, rs7482144 (Xmn1), rs61749494 (*BCL11A*), and rs11759553 (*HMIP*). Details of all 26 samples are available in the Supporting Information document (Table S3). The bold values means the number of TM(thalassemia major) or TI(thalassemia intermedia) patients, with no special significance.

Abbreviations: TI, thalassemia intermedia; TM, thalassemia major.

Molecular screening is an important method for identifying carriers so that we are able to offer counseling and prenatal diagnosis to reduce the birth rate of hemoglobin disorders. There are three groups of variants we considered to be included in the at‐risk assessment for molecular screening. The first group was α‐ or β‐thalassemia variants. The second group was the abnormal hemoglobin variants (Hbs), which may lead to clinical phenotypes, including common variants Hb S (*HBB*:c.20A>T), Hb E (*HBB*:c.79G>A), and rare Hb variants, such as Hb Midnapore (*HBB*:c.161C>T; H. Liu et al., 2016; Panja, Chowdhury, & Basu, 2016; Ware, de Montalembert, Tshilolo, & Abboud, 2017). The benign Hb variants which account for the largest proportion were not included. The third group contained variants resulting in atypical thalassemia (Table 3 and Table S3). For example, compound heterozygotes of variants located in the zinc finger of the *KLF1* gene may lead to microcytic hypochromic anemia (Huang et al., 2015; Perkins et al., 2016). At‐risk assessment is highly recommended for the carriers of these variants.

Here, we used hemoglobinopathies as a model to establish the LOVD‐China variant database and DASH system because hemoglobinopathies are the most common monogenic diseases worldwide and are associated with multiple mutations in disease‐causing genes, as well as modifier genes. The LOVD‐China with DASH system is the first automatic auxiliary diagnosis platform for hemoglobinopathies and thus provides a standard platform for screening, diagnosis, and prevention of hemoglobinopathies. Both these websites will be updated and curated with the increasing production in data by molecular screening, traditional diagnostic approaches, and by the submission of clinicians. We hope that LOVD‐DASH will be a paradigm in the online auxiliary diagnosis of genetic disorders and may be an inspiration for other genetic disorders.

## CONFLICT OF INTEREST

The authors declare that there are no conflict of interest.

## Supporting information

Supporting information

Supporting information
