# Supplementary material for: LOVD–DASH: A comprehensive LOVD database coupled with diagnosis and an at‐risk assessment system for hemoglobinopathies
Source: Hum Mutat. 2019 Sep 11;40(12):2221–9. doi: 10.1002/humu.23863 (PMC6899610; doi:10.1002/humu.23863)

**Supplementary document**

**Supp. Table S1. ACMG classification of reported variants**

All the variants are classified according to ACMG recommendations with the use of specific standard terminology: "pathogenic", "likely pathogenic". "uncertain significance", "likely benign", and "benign".

Table S1 is available as an Excel data file on the journal website.

**Supp. Table S2. The 74 functional variants from 22,309 high-through sequencing data**

Among the 510 β°/β° samples, 65 SNPs and 9 indels were shown to be significant after quantitative trait association analysis in Plink judged the P-values after a Bonferroni correction.

There is bias that SNPs with similar frequencies present some particular linked haplotype.

SNP: single nucleotide polymorphism; Ref: reference allele; Alt: alternative allele.

a The chromosomal locations are given in GRCh37/hg19 coordinates.

b *P*-value calculated for the difference in Hb F levels in the carriers and non-carriers of each SNP.

| **SNPs** | **Locus** | **Region** | **Chr.** | **Location^a^** | **Ref** | **Alt** | **Frequency** | ***P*-value^b^** | **HbF level of carriers(g/L)** | **HbF level of non-carriers(g/L)** |
| --- | --- | --- | --- | --- | --- | --- | --- | --- | --- | --- |
| rs61749494 | *BCL11A* | cds | 2 | 60689441 | T | C | 0.2510 | 4.24×10-6 | 18.6820 | 11.6439 |
| rs10189857 | *BCL11A* | intron | 2 | 60713235 | A | G | 0.9235 | 1.64×10-6 | 12.8591 | 20.0668 |
| rs6545816 | *BCL11A* | intron | 2 | 60714861 | A | C | 0.9216 | 3.24×10-6 | 12.8711 | 19.7456 |
| rs1427407 | *BCL11A* | intron | 2 | 60718043 | T | G | 0.9275 | 1.95×10-6 | 12.9450 | 19.3588 |
| rs7599488 | *BCL11A* | intron | 2 | 60718347 | C | T | 0.9235 | 2.77×10-6 | 12.8542 | 20.1268 |
| rs766432 | *BCL11A* | intron | 2 | 60719970 | C | A | 0.9255 | 1.65×10-5 | 13.0060 | 18.4325 |
| rs4671393 | *BCL11A* | intron | 2 | 60720951 | A | G | 0.9275 | 3.43×10-6 | 12.9880 | 18.8090 |
| rs11759553 | *HMIP* | intergenic | 6 | 135422296 | A | T | 0.3451 | 5.61×10-5 | 17.2904 | 11.3657 |
| rs35959442 | *HMIP* | intergenic | 6 | 135424179 | C | G | 0.3490 | 6.06×10-5 | 17.2321 | 11.3613 |
| rs4895440 | *HMIP* | intergenic | 6 | 135426558 | A | T | 0.3490 | 6.06×10-5 | 17.2321 | 11.3613 |
| rs4895441 | *HMIP* | intergenic | 6 | 135426573 | A | G | 0.3490 | 6.06×10-5 | 17.2321 | 11.3613 |
| rs9402686 | *HMIP* | intergenic | 6 | 135427817 | G | A | 0.3510 | 6.06×10-5 | 17.1452 | 11.3906 |
| rs9494142 | *HMIP* | intergenic | 6 | 135431640 | T | C | 0.3627 | 5.17×10-5 | 16.9296 | 11.4070 |
| rs6934903 | *HMIP* | intergenic | 6 | 135451564 | T | A | 0.3373 | 9.34×10-5 | 16.6348 | 11.7694 |
| rs375867652 | *HMIP* | intergenic | 6 | 135419038 | C | . | 0.3275 | 1.59×10-4 | 17.3037 | 11.5147 |
| rs7936823 | *HBB* | promoter | 11 | 5250168 | G | A | 0.9902 | 3.77×10-8 | 13.1279 | 41.9308 |
| rs6578588 | *_* | intergenic | 11 | 5252251 | T | C | 0.9902 | 7.55×10-9 | 13.1279 | 41.9308 |
| rs3813727 | *HBD* | promoter | 11 | 5255912 | A | G | 0.9902 | 1.93×10-9 | 13.1279 | 41.9308 |
| rs3813726 | *HBD* | promoter | 11 | 5255989 | T | C | 0.0725 | 1.51×10-8 | 25.4724 | 12.4668 |
| rs7948668 | *HBD* | promoter | 11 | 5256647 | A | G | 0.9902 | 3.97×10-8 | 13.1279 | 41.9308 |
| rs3759074 | *HBD* | promoter | 11 | 5257778 | G | A | 0.1000 | 5.45×10-11 | 24.2975 | 12.2006 |
| rs10837643 | *_* | intergenic | 11 | 5258038 | T | A | 0.9902 | 5.18×10-8 | 13.1279 | 41.9308 |
| rs4320977 | *_* | intergenic | 11 | 5258162 | A | G | 0.9902 | 8.61×10-9 | 13.1279 | 41.9308 |
| rs3759073 | *_* | intergenic | 11 | 5258265 | T | C | 0.9882 | 4.47×10-6 | 13.1494 | 35.3273 |
| rs4426158 | *_* | intergenic | 11 | 5258429 | T | C | 0.9902 | 1.24×10-8 | 13.1279 | 41.9308 |
| rs4283007 | *_* | intergenic | 11 | 5258490 | A | T | 0.9902 | 1.55×10-9 | 13.1279 | 41.9308 |
| rs4402323 | *_* | intergenic | 11 | 5258592 | C | T | 0.9902 | 1.32×10-10 | 13.1279 | 41.9308 |
| rs4910543 | *_* | intergenic | 11 | 5258827 | G | C | 0.9902 | 3.41×10-10 | 13.1279 | 41.9308 |
| rs4910735 | *_* | intergenic | 11 | 5258852 | G | A | 0.9902 | 3.41×10-10 | 13.1279 | 41.9308 |
| rs4910544 | *_* | intergenic | 11 | 5258856 | T | A | 0.9902 | 1.55×10-9 | 13.1279 | 41.9308 |
| rs4910736 | *_* | intergenic | 11 | 5258989 | C | A | 0.9902 | 1.32×10-10 | 13.1279 | 41.9308 |
| rs4910737 | *_* | intergenic | 11 | 5259289 | C | T | 0.9882 | 1.41×10-6 | 13.1440 | 35.7790 |
| rs4910738 | *_* | intergenic | 11 | 5259292 | T | A | 0.9882 | 1.14×10-6 | 13.1440 | 35.7790 |
| rs2105819 | *_* | intergenic | 11 | 5259727 | G | C | 0.9902 | 1.87×10-9 | 13.1279 | 41.9308 |
| rs968857 | *_* | intergenic | 11 | 5260458 | T | C | 0.9902 | 2.69×10-7 | 13.1279 | 41.9308 |
| rs968856 | *_* | intergenic | 11 | 5260576 | T | C | 0.9882 | 1.04×10-5 | 13.1411 | 36.0257 |
| rs10768687 | *_* | intergenic | 11 | 5261239 | C | G | 0.9902 | 2.15×10-7 | 13.1279 | 41.9308 |
| rs28379094 | *HBG1* | intron | 11 | 5269806 | C | T | 0.9843 | 1.65×10-12 | 13.0232 | 37.7005 |
| rs2187608 | *HBG1* | intron | 11 | 5269931 | G | C | 0.1373 | 1.41×10-12 | 23.3314 | 11.8320 |
| rs7482933 | *HBG1* | intron | 11 | 5270002 | G | A | 0.8588 | 1.15×10-9 | 11.8743 | 22.7546 |
| rs2855039 | *HBG1* | promoter | 11 | 5271671 | C | T | 0.1373 | 6.93×10-12 | 23.3314 | 11.8320 |
| rs2855038 | *HBG1* | promoter | 11 | 5272154 | T | C | 0.9863 | 4.46×10-11 | 13.1293 | 33.6006 |
| rs2855036 | *HBG1* | promoter | 11 | 5272682 | C | T | 0.1373 | 1.30×10-11 | 23.3314 | 11.8320 |
| rs2855126 | *_* | intergenic | 11 | 5273147 | C | G | 0.9863 | 1.69×10-10 | 13.1293 | 33.6006 |
| rs2255519 | *_* | intergenic | 11 | 5273541 | G | A | 0.9863 | 4.78×10-10 | 13.1293 | 33.6006 |
| rs2855125 | *_* | intergenic | 11 | 5273687 | T | G | 0.9863 | 1.83×10-10 | 13.1293 | 33.6006 |
| rs2236794 | *_* | intergenic | 11 | 5274267 | C | T | 0.9863 | 8.88×10-12 | 13.1293 | 33.6006 |
| rs2070972 | *HBG2* | intron | 11 | 5274717 | A | C | 0.9843 | 5.86×10-11 | 13.1411 | 30.3030 |
| rs11036474 | *HBG2* | intron | 11 | 5275178 | T | C | 0.1412 | 7.90×10-12 | 23.3499 | 11.7764 |
| rs11036475 | *HBG2* | intron | 11 | 5275240 | G | A | 0.9863 | 2.06×10-11 | 13.1293 | 33.6006 |
| rs11036476 | *HBG2* | intron | 11 | 5275343 | C | T | 0.9863 | 9.69×10-11 | 13.1293 | 33.6006 |
| rs2070973 | *HBG2* | intron | 11 | 5275407 | T | C | 0.9863 | 9.69×10-11 | 13.1293 | 33.6006 |
| rs7482144 | *HBG2* | promoter | 11 | 5276169 | G | A | 0.1412 | 1.81×10-11 | 23.3499 | 11.7764 |
| rs2855123 | *HBG2* | promoter | 11 | 5277078 | A | T | 0.9863 | 3.92×10-10 | 13.1293 | 33.6006 |
| rs2855122 | *HBG2* | promoter | 11 | 5277236 | C | T | 0.9863 | 1.20×10-11 | 13.1293 | 33.6006 |
| rs2855121 | *HBG2* | promoter | 11 | 5277291 | C | T | 0.1392 | 7.90×10-12 | 23.3682 | 11.7998 |
| rs11036496 | *_* | intergenic | 11 | 5280022 | G | C | 0.9863 | 1.18×10-11 | 13.1293 | 33.6006 |
| rs72872549 | *HBE1* | intron | 11 | 5290053 | C | T | 0.1392 | 7.90×10-12 | 23.3682 | 11.7998 |
| rs67385638 | *HBE1* | intron | 11 | 5290370 | C | G | 0.1412 | 7.90×10-12 | 23.3499 | 11.7764 |
| rs3759071 | *HBE1* | promoter | 11 | 5291532 | G | A | 0.9863 | 1.52×10-10 | 13.1293 | 33.6006 |
| rs3759070 | *HBE1* | promoter | 11 | 5291628 | C | G | 0.1392 | 4.18×10-12 | 23.3682 | 11.7998 |
| rs3759069 | *HBE1* | promoter | 11 | 5291830 | A | G | 0.9863 | 3.92×10-10 | 13.1293 | 33.6006 |
| rs10768737 | *HBE1* | promoter | 11 | 5291872 | T | C | 0.9863 | 5.86×10-11 | 13.1293 | 33.6006 |
| rs114883746 | *HBB-LCR* | intergenic | 11 | 5297582 | A | C | 0.0824 | 2.32×10-8 | 27.5757 | 12.1391 |
| rs138186609 | *HBB-LCR* | intergenic | 11 | 5301648 | G | A | 0.0510 | 4.27×10-5 | 24.6996 | 12.8039 |
| rs10665762 | *_* | intergenic | 11 | 5259103 | . | AC | 0.9902 | 8.40×10-10 | 13.1279 | 41.9308 |
| rs78981054 | *HBG1* | intron | 11 | 5270347 | AAAG | . | 0.9863 | 4.66×10-8 | 13.1293 | 33.6006 |
| rs34306743 | *HBG1* | promoter | 11 | 5272553 | . | A | 0.1373 | 1.30×10-11 | 23.3314 | 11.8320 |
| rs34879481 | *HBG2* | 3’UTR | 11 | 5274452 | . | T | 0.1392 | 4.19×10-12 | 23.3682 | 11.7998 |
| rs34501951 | *_* | intergenic | 11 | 5280840 | A | . | 0.1373 | 5.88×10-12 | 23.5266 | 11.8009 |
| rs3834466 | *HBE1* | promoter | 11 | 5291563 | . | T | 0.9863 | 2.06×10-11 | 13.1293 | 33.6006 |
| rs5789387 | *_* | intergenic | 11 | 5294135 | T | . | 0.9471 | 6.07×10-5 | 12.9911 | 20.9089 |
| rs62030346 | *_* | intergenic | 16 | 213795 | T | C | 0.0314 | 2.87×10-5 | 21.8756 | 13.1361 |
| rs483352838 | *KLF1* | cds | 19 | 12996518 | . | GGCGCCG | 0.0137 | 1.93×10-6 | 39.2343 | 13.0509 |

**Supp. Table S3. Phenotype-genotype dataset of 26 complicated cases**

TM: Thalassemia major, TI: Thalassemia Intermedia

Hb: Hemoglobin; MCV: mean cell volume; MCH: mean corpuscular hemoglobin content. Hb, MCH, and MCV were measured on an automated hematology analyzer. HbF and HbA_2_ were detected by high-performance liquid chromatography (HPLC) and capillary electrophoresis (CE). Genotypes were analyzed by NGS.

#ID is corresponding individual ID in LOVD3.0 database. (http://www.genomed.zju.edu.cn/LOVD3/individuals)

*HGVS nomenclature mentioned in table :

ααα^anti3.7^ NC_000016.9:g.223300_227103dup

ααα^anti4.2^ NC_000016.9:g.219817_224074dup

3.7 NC_000016.9:g.223300_227103del

CD122(WS) NM_000517.4:c.369C>G

CD17 NM_000518.4:c.52A>T

CD41-42 NM_000518.4:c.126_129del

-28C NM_000518.4:c.-78A>C

IVS-II-654 NM_000518.4:c.316-197C>T

CD30 NM_000518.4:c.91A>C

IVS-I-1 NM_000518.4:c.92+1G>T

Chinese NC_000011.9:g.5191148_5270051del

CD43 NM_000518.4:c.130G>T

| **ID^#^** | **Major classes** | **Age**  **(year)** | **Gender** | **Hb**  **(g/l)** | **MCV(fL)** | **MCH**  **(pg)** | **HbF%** | **HbA_2_**  **(+E)%** | **Age of onset (months)** | **Transfusion times per year** | **α-genotype*** | **β-genotype*** | **Modifiers** | **Clinical phenotype** |
| --- | --- | --- | --- | --- | --- | --- | --- | --- | --- | --- | --- | --- | --- | --- |
| 00002089 | α-globin gene triplication | 25 | F | 100 | 53.6 | 17.4 | 0.3 | 5.7 |  |  | ααα^anti3.7^/αα | CD17/N | / | TI |
| 00002090 | α-globin gene triplication | 24 | M | 101 | 62 | 18.6 | 1 | 6.1 |  |  | ααα^anti4.2^/αα | CD17/N | / | TI |
| 00002091 | α-globin gene triplication | 28 | F | 124 | 60 | 18.9 | 2 | 5.6 |  |  | ααα^anti4.2^/αα | CD41-42/N | / | TI |
| 00002092 | α-globin gene triplication | 28 | F | 109 | 60.3 | 19.5 | 1 | 5 |  |  | ααα^anti4.2^/αα | CD41-42/N | / | TI |
| 00002093 | α-globin gene triplication | 25 | M | 118 | 62.7 | 19.2 | 0.4 | 4.9 |  |  | ααα^anti4.2^/αα | CD41-42/N | / | TI |
| 00002094 | α-globin gene triplication | 25 | F | 83 | 63.1 | 20.1 | 1.4 | 4.8 |  |  | ααα^anti4.2^/αα | CD17/N | / | TI |
| 00001989 | α-globin gene triplication |  | F | 97 | 80.1 | 26.8 | 1.9 | 3.2 |  |  | ααα^anti3.7^/ααα^anti4.2^ | CD41-42/N | / | TI |
| 00001994 | α-globin gene triplication |  | F | 99 | 63.9 | 19.2 | 1.8 | 5.4 |  |  | ααα^anti3.7^/αα | CD41-42/N | / | TI |
| 00001995 | α-globin gene triplication |  | M | 108 | 67.7 | 20.6 | 1.6 | 5.3 |  |  | ααα^anti3.7^/αα | CD41-42/N | / | TI |
| 00001474 | α-globin gene triplication | 9 | M | 101 | 83 | 27.2 | 18.2 | 2.6 | 3 | 12 | ααα^anti3.7^/αα | CD41-42/-28C | / | TM |
| 00000245 | α-globin gene triplication |  | M | 80 | 79.7 | 26.1 |  |  |  |  | ααα^anti3.7^/αα | CD41-42/IVS-II-654 | / | TM |
| 00001254 | α-globin gene triplication | 9 | M | 95 | 79 | 26.3 | 4.7 | 2.6 | 5 | 12 | ααα^anti4.2^/αα | CD41-42/CD41-42 | / | TM |
| 00001325 | α-globin gene triplication | 6 | F | 80 | 86 | 25.9 | 2.8 | 2.6 | 3 | 15 | ααα^anti3.7^/αα | CD41-42/CD41-42 | / | TM |
| 00000120 | non-globin modifier genes |  |  | 90 | 73 | 22.5 | 56.6 | 4 | 48 | 12 | N/N | CD41-42/CD17 | KLF1^M^/KLF1^N^ | TI |
| 00000425 | non-globin modifier genes |  |  | 83 | 61 | 20 | 48.1 | 4.7 | 24 | 4 | N/N | CD41-42/CD17 | KLF1^M^/KLF1^N^ | TI |
| 00001189 | non-globin modifier genes |  |  | 91 | 72 | 23.9 | 13.2 | 3.4 | 36 | 12 | N/N | CD17/CD17 | KLF1^M^/KLF1^N^ | TI |
| 00001338 | non-globin modifier genes |  |  | 86 | 62 | 20.5 | 44.7 | 3.5 | 36 | 12 | N/N | CD41-42/CD41-42 | KLF1^M^/KLF1^N^ | TI |
| 00002098 | non-globin modifier genes |  |  | 71 | 107 | 27.3 | 94.3 | 3.9 | 31 | 1 | N/N | CD41-42/CD30 | KLF1^M^/KLF1^N^ | TI |
| 00000223 | non-globin modifier genes |  |  | 83 | 73 | 22.2 | 80 | 1.2 | 180 | 2 | 3.7/N | Chinese/CD17 | 4 significant variants | TI |
| 00001490 | non-globin modifier genes |  |  | 34 | 75 | 22.3 | 41.2 | 1.9 | 36 | 6 | CD122(WS)/N | CD41-42/CD17 | 4 significant variants | TI |
| 00001630 | non-globin modifier genes |  |  | 64 | 74 | 23.1 | 79.2 | 3.4 | 72 | 1 | SEA/N | CD43/CD17 | 4 significant variants | TI |
| 00001677 | non-globin modifier genes |  |  | 81 | 66 | 19.7 | 84.2 | 3.9 | 96 | 0 | SEA/N | CD41-42/IVS-I-1 | 4 significant variants | TI |
| 00002099 | non-globin modifier genes |  |  | 72 | 68 | 19.9 | 13.8 | 3.6 | 72 | 12 | SEA/N | CD41-42/CD41-42 | 4 significant variants | TI |
| 00002095 | Microcytic hypochromic anemia | 25 | M | 117 | 89.2 | 26.7 | 18.8 | 3.2 |  |  | N/N | N/N | KLF1^M^/KLF1^M^ | TI |
| 00002096 | Microcytic hypochromic anemia | 4 | M | 79 | 72.4 | 24.9 | 26.6 | 4.8 |  |  | N/N | N/N | KLF1^M^/KLF1^M^ | TI |
| 00002097 | Microcytic hypochromic anemia | 12 | M | 97 | 72.4 | 25.1 | 33.2 | 3.9 |  |  | N/N | N/N | KLF1^M^/KLF1^M^ | TI |

**Supp. Figure S1.** **The flowchart to identify the functional variants exerting significant impact on clinical severity of β thalassemia patients.**

Among the 2,087 hemoglobinopathies samples, 510 β^0^/β^0^ samples with complete phenotype and genotype information were selected to perform quantitative trait association analysis in Plink. After Bonferroni correction, 74 variants were identified as functional modifier mutations. The analysis did not eliminate the effect of gender and age factors.

The command line used for quantitative trait association analysis was

plink --noweb --file mydata --assoc --out HbF_association


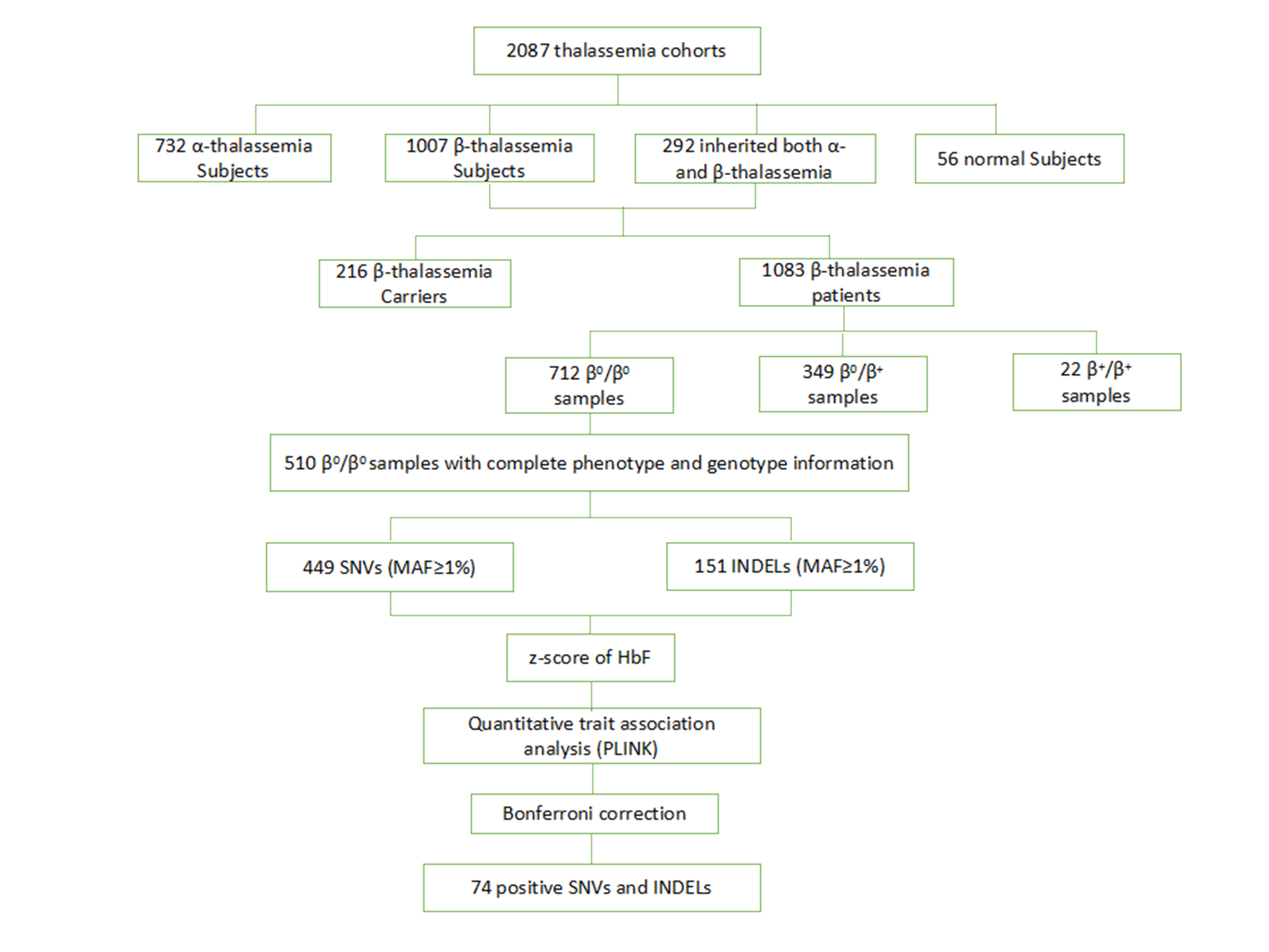


**Supp. Figure S2. Diagnostic flowchart for identification of thalassemia carriers from the phenotype.**

MCH: mean corpuscular hemoglobin content; MCV: mean cell volume; thal: thalassemia; Ref: reference value. MCH and MCV were measured on an automated hematology analyzer. HbF and HbA_2_ were detected by high-performance liquid chromatography (HPLC) and capillary electrophoresis (CE). According to the standard used in the clinic, the reference value of Hb A_2_ was as follows: 4.0% in HPLC (Biorad); 3.5% in CE (Sebia); and 6.0% in CE (Helena).


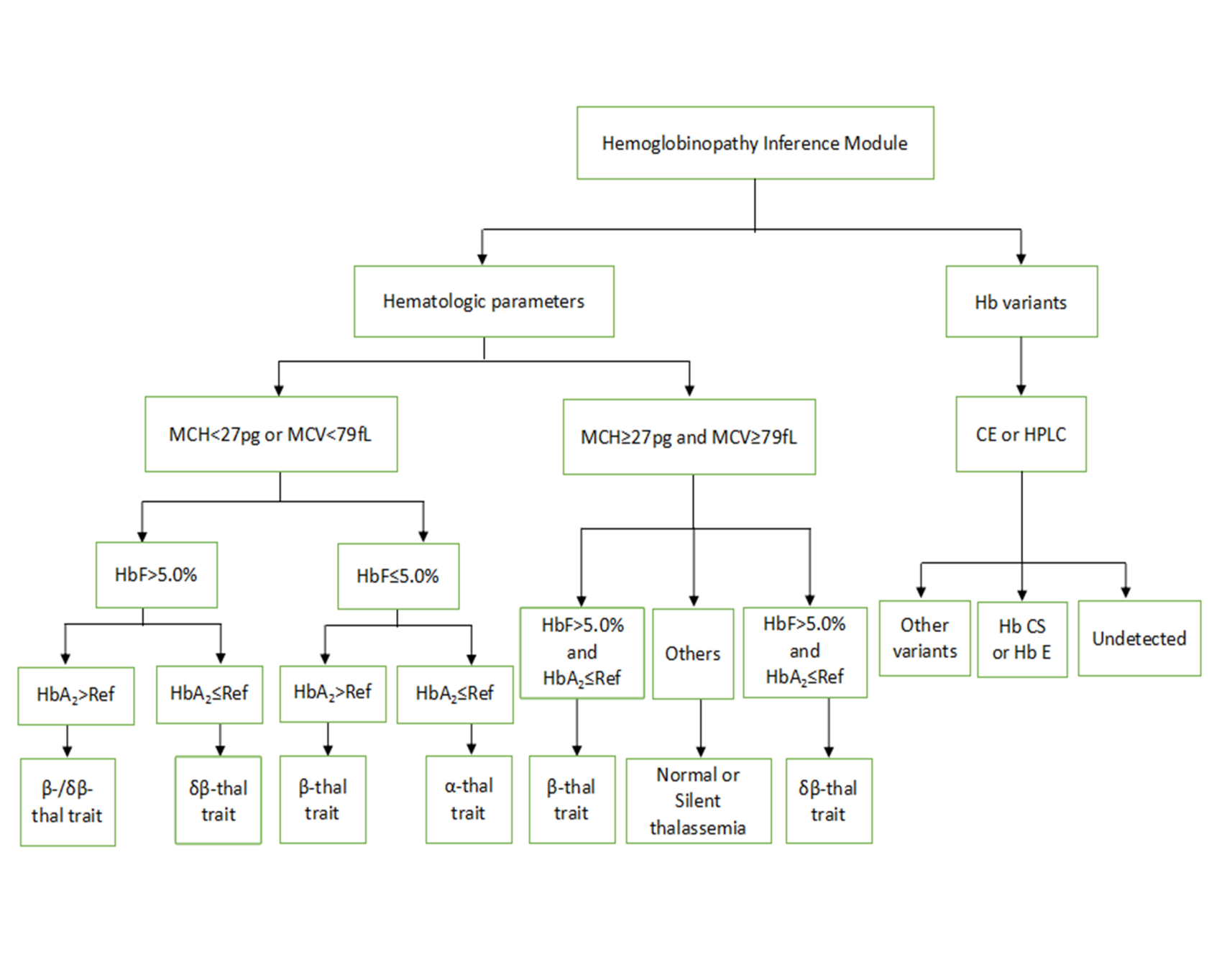

Supplement: Supplementary file 2 — Supporting information [file HUMU-40-2221-s002.docx]
